# Microbial Abundances Predict Methane and Nitrous Oxide Fluxes from a Windrow Composting System

**DOI:** 10.3389/fmicb.2017.00409

**Published:** 2017-03-20

**Authors:** Shuqing Li, Lina Song, Xiang Gao, Yaguo Jin, Shuwei Liu, Qirong Shen, Jianwen Zou

**Affiliations:** ^1^Jiangsu Key Laboratory of Low Carbon Agriculture and GHGs Mitigation, College of Resources and Environmental Sciences, Nanjing Agricultural UniversityNanjing, China; ^2^Jiangsu Key Laboratory and Engineering Center for Solid Organic Waste Utilization, Jiangsu Collaborative Innovation Center for Solid Organic Waste Resource Utilization, Nanjing Agricultural UniversityNanjing, China

**Keywords:** CH_4_, carbon and nitrogen biogeochemistry, N_2_O, bacterial gene abundance, greenhouse gas, statistical model

## Abstract

Manure composting is a significant source of atmospheric methane (CH_4_) and nitrous oxide (N_2_O) that are two potent greenhouse gases. The CH_4_ and N_2_O fluxes are mediated by methanogens and methanotrophs, nitrifying and denitrifying bacteria in composting manure, respectively, while these specific bacterial functional groups may interplay in CH_4_ and N_2_O emissions during manure composting. To test the hypothesis that bacterial functional gene abundances regulate greenhouse gas fluxes in windrow composting systems, CH_4_ and N_2_O fluxes were simultaneously measured using the chamber method, and molecular techniques were used to quantify the abundances of CH_4_-related functional genes (*mcrA* and *pmoA* genes) and N_2_O-related functional genes (*amoA*, *narG*, *nirK*, *nirS*, *norB*, and *nosZ* genes). The results indicate that changes in interacting physicochemical parameters in the pile shaped the dynamics of bacterial functional gene abundances. The CH_4_ and N_2_O fluxes were correlated with abundances of specific compositional genes in bacterial community. The stepwise regression statistics selected pile temperature, *mcrA* and NH_4_^+^ together as the best predictors for CH_4_ fluxes, and the model integrating *nirK*, *nosZ* with *pmoA* gene abundances can almost fully explain the dynamics of N_2_O fluxes over windrow composting. The simulated models were tested against measurements in paddy rice cropping systems, indicating that the models can also be applicable to predicting the response of CH_4_ and N_2_O fluxes to elevated atmospheric CO_2_ concentration and rising temperature. Microbial abundances could be included as indicators in the current carbon and nitrogen biogeochemical models.

## Introduction

It is of great concern worldwide that gaseous emissions from management of organic solid waste contribute to regional and global-scale environmental processes, such as eutrophication, acidification, and climate change (Naylor et al., 2005; Hou et al., 2015; Owen and Silver, 2015; Pardo et al., 2015). Organic waste management has been identified as an important source of anthropogenic greenhouse gas (GHG) emissions, particularly methane (CH_4_) and nitrous oxide (N_2_O) [Intergovernmental Panel on Climate Change (IPCC), 2006]. Global CH_4_ and N_2_O emissions contribute considerably to the radiative forcing of the atmosphere, as their global warming potentials are 298 and 25 times that of carbon dioxide (CO_2_) on mass basis over the 100-year time horizon, respectively [Intergovernmental Panel on Climate Change (IPCC), 2013]. Manures from livestock production account for 30–50% of the global agricultural N_2_O emissions and 12–41% of the total agricultural CH_4_ emissions for most countries (Oenema and Tamminga, 2005; Chadwick et al., 2011).

Manure composting is an alternative agricultural strategy for organic waste management that produces organic fertilizer to improve soil structure and fertility in croplands (Larney and Hao, 2007). The CH_4_ and N_2_O fluxes from manure composting have been studied extensively, contributing to a comprehensive assessment of CH_4_ and N_2_O emissions from manure composting worldwide [Czepiel et al., 1996; Hao et al., 2001; Intergovernmental Panel on Climate Change (IPCC), 2006; Xu et al., 2007; Mulbry and Ahn, 2014; Jiang et al., 2015; Pardo et al., 2015]. Yet, little is known about the interaction between pile physicochemical parameters and bacterial community, which has a key role in CH_4_ and N_2_O emissions from manure composting (Sharma et al., 2011; Angnes et al., 2013; Zhang et al., 2015). In particular, few studies have simultaneously focused on quantitative analysis of bacterial community composition and CH_4_ and N_2_O fluxes from composting manure (Maeda et al., 2010a,b; Chen et al., 2014; Zhang et al., 2015). Comparative quantitative analysis of specific bacterial functional groups and their interplay in CH_4_ and N_2_O emissions during manure composting are still limited.

To date, genes encoding enzymes involved in CH_4_ and N_2_O emissions have been targets of choice for studies focusing on functional groups of bacteria. This focus is fundamental for understanding mechanisms of carbon and nitrogen biogeochemistry and strategies for GHGs mitigation (Hu et al., 2015). Morales et al. (2010) illustrated denitrifying gene abundances as proxies for predicting N_2_O emissions from soils as a response to different long-term land management regimes. Regan et al. (2011) found the evidence that differences in microbial abundances can help explain enhanced N_2_O emissions in permanent grasslands under elevated atmospheric carbon dioxide. Nevertheless, a trade-off between CH_4_ and N_2_O fluxes has frequently been found in rice paddies and manure composting (Hou et al., 2001; Zou et al., 2005; Ahn et al., 2011; Shen et al., 2011; Mulbry and Ahn, 2014), simultaneous comparisons of the abundance of multiple CH_4_- and N_2_O-related genes and their interactions would be highly needed, especially when targeting functional bacteria to mitigate GHGs emission from agriculture (Hu et al., 2015).

Manure composting system is suggested as a good study model to examine the role of microbial abundances in shaping dynamics of CH_4_ and N_2_O fluxes due to their sensitive responses to changes in pile physicochemical properties, nitrogen transformation, and organic carbon decomposition during composting (Chadwick et al., 2011). Methane is produced by methanogenic organisms during the anaerobic degradation of organic materials; and the final key step, being reduction of CO_2_ using H_2_ to generate CH_4_, is catalyzed by methyl-coenzyme M reductase (MCR, EC 2.8.4.1) (Kim et al., 2008). The highly conserved *mcrA* gene encoding the α-subunit of MCR has been widely used for analysis and quantification of methanogen communities (Pereyra et al., 2010; Sonoki et al., 2013). The generated CH_4_ could be oxidized to methanol with the catalysis of particulate membrane bound methane monooxygenase (EC 1.14.13.25) (Xin et al., 2004). The *pmoA* gene encoding the α-subunit is widely used as the indicator for quantification of the methanotrophs from environmental samples (Wasmund et al., 2009; Sharma et al., 2011). The CH_4_ flux is the net outcome and combined action of methanogen and methanotrophs that are closely related to changes in physicochemical parameters and environmental factors during manure composting process (Sonoki et al., 2013).

During manure composting, NH_4_^+^ generated from amino acids can be oxidized to NO_2_^-^ by ammonia-oxidizing archaea and bacteria (AOA and AOB, respectively), through ammonia monooxygenase (EC 1.14.99.39, encoding by *amoA*) and hydroxylamine oxidoreductase. A part of the NO_2_^-^ could be oxidized to NO_3_^-^ by nitrite-oxidizing bacteria (NOB) (Maeda et al., 2011). Bacterial denitrification is a biochemical reaction where oxidized forms of nitrogen, including nitrate, nitrite, nitric oxide, and nitrous oxide, are gradually reduced (Wang et al., 2013). The four steps are generally catalyzed by nitrate reductase (encoding by *narG*), nitrite reductase (*nirS/nirK*), nitric oxide reductase (*norB*), and nitrous oxide reductase (*nosZ*) (Maeda et al., 2011). Eventually, the N_2_O emission is a result of dynamic balance between N_2_O production and consumption. In addition, given that a trade-off between CH_4_ and N_2_O fluxes has been well documented in windrow composting systems (Hao et al., 2001; Zou et al., 2005; Ahn et al., 2011; Shen et al., 2011; Mulbry and Ahn, 2014), some compositional bacterial genes could be multifunctional as proxies for indicating dynamics of CH_4_ and N_2_O fluxes during windrow composting.

We conducted an *in situ* measurement of CH_4_ and N_2_O fluxes from a commercial composting windrow. Molecular techniques were used to quantify the abundances of CH_4_- and N_2_O-related functional genes. The main objective of this study is to examine whether bacterial gene abundances can indicate dynamics of CH_4_ and N_2_O fluxes during windrow composting. Specifically, we aimed to test three general hypotheses. The first hypothesis stated that changes in pile physicochemical parameters would shape diverse time course patterns of bacterial functional genes abundance during windrow composting, given that bacterial community response variables are sensitive to environmental change. Second, we predicted that some specific physicochemical parameters and compositional bacterial enzymes encoded by relevant genes would be multifunctional as involved both in CH_4_ and N_2_O due to a trade-off between CH_4_ and N_2_O fluxes during windrow composting. Eventually, as both production and consumption of CH_4_ and N_2_O are controlled by the interplay of enzyme encoding bacterial functional genes, we hypothesized that bacterial functional gene abundances could be used as proxies for indicating dynamics of CH_4_ and N_2_O fluxes during windrow composting.

## Materials and Methods

### Windrow Composting Construction

The windrow composting experiment was carried out in a commercial organic fertilizer company (Jiangyin Lianye Biological Science and Technology Co., Ltd.), located at Wuxi, Jiangsu province, China. The experiment was initiated on April 26 and completed on June 29, 2014 (65 days). Three replicate commercial-scale compost piles were constructed using a mixture of dairy manure solids and straw bedding with a mixing ratio of 80%:20% on a fresh weight basis. Sawdust used for dairy manure solids was obtained from scraped dairy manure with 75% moisture in a local dairy feedlot. Chopped rice straw collected from local paddy rice fields was used as a bulking agent and a source of carbon. Uniform rectangle windrows were placed on individual platforms to enable determination of windrow mass values in an open-sided but roofed compost. The volume of each compost windrow was set up to be approximately 125 m^3^ (40 m in length, 2.8 m in width, and 1.1 in height). The composting process can be generally divided into two phases, namely, the bio-oxidative phase with mechanical turning for 25 days (Phase I, April 26 to May 20, 2014) and the cooling and maturing phase without pile turning for 40 days (Phase II, May 21 to June 29, 2014) (Chen et al., 2014). The windrow was mechanically turned using an astraddle compost turner once every 2 days during Phase I, and thereafter the compost piles were moved away for post-maturation without turning. Three compost windrows were treated as experimental replicates. Each windrow along its length was sub-divided into three sections that were treated as three parallel locations for substrate material and gas sampling.

### Measurement of CH_4_ and N_2_O Fluxes

The fluxes of CH_4_ and N_2_O during compositing were simultaneously measured using vented chamber technique (Hao et al., 2001; Xin et al., 2004; Zou et al., 2005; Chen et al., 2014). Gas samples were taken once a week except supplementary sampling episodes as needed to capture high flux peaks. To stabilize the disturbance, PVC chamber collar bases (30 cm length × 30 cm width × 25 cm height) were pre-inserted 25 cm into the pile 10–12 h before each gas sampling. The top edge of the collar base exhibits a groove (5 cm in depth) that can be filled with water to seal the rim of the chamber during gas sampling. While taking gas samples, the opaque chamber (30 cm length × 30 cm width × 50 cm height) was placed on the peak of each windrow with rim of the chamber fitted into the groove of collar. Gas samples were extracted from inside the chambers using 60-mL plastic syringes fitted with three-way stopcocks at 0, 5, 10, 20, and 30 min after chamber closure and immediately injected into a 50 mL pre-evacuated Exetainer fitted with butyl rubber septa (Hao et al., 2001; Chen et al., 2014). Gas samples were taken between 0800 and 1000 LST on each sampling day, and they were transported to the laboratory for analysis by gas chromatograph (GC) within a few hours (Zou et al., 2009; Liu et al., 2010).

The mixing ratios of CH_4_ and N_2_O were analyzed with a modified GH (Agilent 7890) equipped with a flame ionization detector (FID) and an electron capture detector (ECD) (Zou et al., 2005; Liu et al., 2012). A non-linear fitting approach was adopted to determine the CH_4_ and N_2_O fluxes (Hao et al., 2001; Kroon et al., 2008; Chen et al., 2014). On each sampling day, mean of fluxes taken from three parallel sections within each windrow represent flux measurement of the sampling windrows. Average fluxes and standard deviations of CH_4_ and N_2_O were calculated from three replicate windrows. Accumulative CH_4_ and N_2_O emissions during compositing were sequentially accumulated from the fluxes between every two adjacent intervals of measurements (Zou et al., 2005; Liu et al., 2012).

### Real-Time Quantitative PCR (qPCR) Assays of the Functional Genes

Real-time quantitative PCR (qPCR) was performed for investigation of the functional microbial community dynamics during the composting process (days 4, 10, 16, 25, 37, 46, 55, 61, and 65). The genes encoding the key enzymes involved in CH_4_ and N_2_O emissions included α-subunit of methyl-coenzyme M reductase (*mcrA*), α-subunit methane monooxygenase (*pmoA*), ammonia monooxygenase (*amoA*), nitrate reductase (*narG*), nitrite reductase (*nirK* and *nirS*), nitric oxide reductase (*norB*), and nitrous oxide reductase (*nosZ*). The information of these functional genes and the using primers and conditions were referenced in **Table 1**. According to the manufacturer’s instructions, DNA was extracted from the compost samples that stored at -80°C using the UltraClean soil DNA isolation kit (Mo Bio, USA). Each DNA sample for next-analysis was the mixture of DNA extractions from three parallel sections within each windrow. The concentrations of DNA samples were determined by a Nanodrop (Thermo Scientific, USA). The amplified fragments for each functional gene based on different primers (**Table 1**) were cloned in pMD 18-T vector and sequenced, the correct clones corresponding to each functional gene were stepwise 10-fold diluted for standard curve preparation. The qPCR amplifications were performed in a total volume of 20 μL using a SYBR@ Premix Ex Taq^TM^ (Takara, China), with reaction mixture consisting of 10 μL SYBR@ Premix Ex Taq, 0.4 μL each primer^TM^ (10 μmol L^-1^), 0.4 μL ROX reference dye II (50×), 2 μL template DNA, and 6.8 μL sterile water. Amplification was performed triplicate using 7500 System (ABI, USA). The detailed reaction conditions were listed in **Table 1**. Fluorescence normalization and data analysis were performed with 7500 Fast System SDS software (ABI, USA).

**Table 1:** The primers used for quantitative PCR in this study.

Gene	Name	Sequence	Thermal profile	No. cycles	Product size	Reference
*mcrA*	mlas	GGTGGTGTMGGDTTCACMCARTA	30 s-95°C, 95°C-15 s, 55°C-30 s,72°C-30 s, 80°C-30 s	1	509 bp	Steinberg and Regan, 2009
	*mcr*-rev	CGTTCATBGCGTAGTTVGGRTAGT	95°C-5 s, 60°C-34 s, 72°C-15 s 95°C-15 s, 55°C-30 s,72°C-30 s, 80°C-30 s	40		
*pmoA*	*pmoA*189-f	GGNGACTGGGACTTCTGG	30 s-95°C, 95°C-15 s, 55°C-30 s,72°C-30 s, 80°C-30 s	1	472 bp	Costello and Lidstrom, 1999
	mb661-r	CCGGMGCAACGTCYTTACC	95°C-5 s, 60°C-34 s, 72°C-15 s 95°C-15 s, 55°C-30 s,72°C-30 s, 80°C-30 s	40		
*amoA*	*amoA*-1F	GGGGTTTCTACTGGTGGT	30 s-95°C,95°C-15 s, 55°C-30 s,72°C-30 s, 80°C-30 s	1	491 bp	Rotthauwe et al., 1997
	*amoA*-2R	CCCCTCKGSAAAGCCTTCTTC	95°C-5 s, 55°C-34 s, 72°C-15 s 95°C-15 s, 55°C-30 s,72°C-30 s, 80°C-30 s	40		
*narG*	*narG*-1960m2f	TAYGTSGGGCAGGARAAACTG	30 s-95°C, 95°C-15 s, 55°C-30 s,72°C-30 s, 80°C-30 s	1	110 bp	López-Gutiérrez et al., 2004
	*narG*-2050m2r	CGTAGAAGAAGCTGGTGCTGTT	95°C-5 s, 60°C-34 s, 72°C-15 s 95°C-15 s, 55°C-30 s,72°C-30 s, 80°C-30 s	40		
*nirS*	*nirSCd3aF*	TACCACCCSGARCCGCGCGT	30 s-95°C,95°C-15 s, 55°C-30 s,72°C-30 s, 80°C-30 s	1	425 bp	Braker et al., 1998
	*nirS*R3cd	GCCGCCGTCRTGVAGGAA	95°C-5 s, 58°C-34 s, 72°C-15 s 95°C-15 s, 55°C-30 s,72°C-30 s, 80°C-30 s	40		
*nirK*	*nirK*F1aCu	ATCATGGTSCTGCCGCG	30 s-95°C, 95°C-15 s, 55°C-30 s,72°C-30 s, 80°C-30 s	1	473 bp	Henry et al., 2004
	*nirK*R3Cu	GCCTCGATCAGRTTGTGGTT	95°C-5 s, 58°C-34 s, 72°C-15 s 95°C-15 s, 55°C-30 s,72°C-30 s, 80°C-30 s	40		
*norB*	qnorB2F	GGNCAYCARGGNTAYGA	30 s-95°C, 95°C-15 s, 55°C-30 s,72°C-30 s, 80°C-30 s	1	262 bp	Braker and Tiedje, 2003
	qnorB5R	ACCCANAGRTGNACNACCCACCA	95°C-5 s, 60°C-34 s, 72°C-15 s 95°C-15 s, 55°C-30 s,72°C-30 s, 80°C-30 s	40		
*nosZ*	*nosZ*-F	AGAACGACCAGCTGATCGACA	30 s-95°C, 95°C-15 s, 55°C-30 s,72°C-30 s, 80°C-30 s	1	300 bp	Scala and Kerkhof, 1998
	*nosZ*-R	TCCATGGTGACGCCGTGGTTG	95°C-5 s, 60°C-34 s, 72°C-15 s 95°C-15 s, 55°C-30 s,72°C-30 s, 80°C-30 s	40		

*M = A/C, D = A/G/T, R = A/G, B = C/G/T, V = G/A/C, N = A/C/G/T, Y = C/T, K = G/T, S = G/C*.

### Physicochemical Parameters Determination

Windrow temperature was measured by inserting the mercury thermometers at 30 cm depth of the pile on each gas sampling day. To examine dynamics of physicochemical parameters of composting material, manure material samples were taken while gas flux sampling (**Table 2**). Samples were randomly collected from three longitudinal sections and mixture, generating approximately 300 g of subsamples. The collected samples were divided into three parts, two parts were immediately preserved at 4°C until analysis, while the other part was air-dried, passed through a 0.15 mm sieve, and stored in a desiccator as needed for further analysis. The moisture content of different fresh samples was determined by oven-drying to a constant weight at 105°C. The C/N ratio was calculated based on the total organic carbon (TOC) and total nitrogen (TN) contents that were determined by an auto elemental analyzer (Vario EL III, Elementar, Germany). For analysis of the water-soluble fractions of the composting material, the aqueous compost extracts were obtained by shaking of the mixture of 20 g of fresh sample with 200 mL distilled water (1:10 w/v ratio) on a horizontal shaker at 25°C, as described in Castaldi et al. (2008). The pH was performed on aqueous suspensions of the fresh samples (1:10, w/v, compost/water ratio) using a pH electrode (PHS-3C mv/pH detector, Shanghai, China). The NH_4_^+^-N, NO_3_^-^-N, and NO_2_^-^-N of composting material were extracted with 100 ml 2 M KCl solution at a ratio of 1:20 at 25°C and measured following the three wavelength ultraviolet spectrometry and indophenol blue method, using the ultraviolet spectrophotometer, respectively (HITACHI, U-2900, Japan).

**Table 2:** Changes of physicochemical parameters (mean ± SE, *n* = 3) during windrow dairy manure composting.

Days	Temperature (°C)	Moisture (%)	pH	SOC (%)	TN (%)	C/N	NH_4_^+^ (g⋅kg^-1^ DM)	NO_3_^-^ (g⋅kg^-1^ DM)	NO_2_^-^ (mg⋅kg^-1^ DM)
2	44.7 ± 0.1	60.1 ± 0.9	8.15 ± 0.18	23.7 ± 0.3	1.45 ± 0.11	16.4 ± 1.3	2.40 ± 0.29	0.37 ± 0.02	1.89 ± 0.10
4	53.8 ± 0.0	57.1 ± 1.3	8.05 ± 0.01	23.2 ± 0.8	1.52 ± 0.06	15.3 ± 0.2	2.31 ± 0.33	0.28 ± 0.02	1.13 ± 0.18
6	67.7 ± 0.3	52.1 ± 1.4	8.34 ± 0.03	21.3 ± 1.0	1.35 ± 0.09	15.7 ± 0.2	1.40 ± 0.09	0.40 ± 0.03	1.73 ± 0.05
10	68.0 ± 0.1	48.0 ± 1.2	8.10 ± 0.03	22.6 ± 0.3	1.35 ± 0.01	16.7 ± 0.2	1.34 ± 0.58	0.36 ± 0.01	1.60 ± 0.06
16	65.4 ± 0.3	48.5 ± 1.4	8.06 ± 0.10	22.2 ± 0.7	1.42 ± 0.05	15.7 ± 1.1	2.02 ± 0.01	0.32 ± 0.01	1.26 ± 0.08
25	63.7 ± 0.4	43.1 ± 4.7	7.93 ± 0.01	21.3 ± 0.2	1.53 ± 0.00	13.9 ± 0.2	1.44 ± 0.00	0.42 ± 0.05	1.54 ± 0.16
37	55.9 ± 0.3	28.4 ± 0.5	7.96 ± 0.03	20.3 ± 0.2	1.61 ± 0.03	12.7 ± 0.1	1.48 ± 0.07	0.32 ± 0.00	2.02 ± 0.08
46	55.5 ± 0.0	16.5 ± 0.8	8.11 ± 0.01	21.5 ± 0.2	1.56 ± 0.06	13.8 ± 0.6	1.49 ± 0.02	0.36 ± 0.00	1.51 ± 0.25
52	49.6 ± 0.1	21.4 ± 0.5	8.12 ± 0.03	20.8 ± 0.6	1.63 ± 0.01	12.7 ± 0.3	1.38 ± 0.05	0.38 ± 0.04	1.60 ± 0.23
55	48.1 ± 0.0	18.9 ± 1.7	8.02 ± 0.02	20.4 ± 0.6	1.63 ± 0.05	12.5 ± 0.1	0.99 ± 0.06	0.46 ± 0.03	1.49 ± 0.14
61	46.0 ± 0.1	13.5 ± 0.0	8.16 ± 0.02	21.4 ± 0.2	1.61 ± 0.01	13.3 ± 0.2	1.09 ± 0.21	0.34 ± 0.00	1.59 ± 0.08
65	45.2 ± 0.2	23.7 ± 0.0	7.99 ± 0.04	20.8 ± 0.0	1.65 ± 0.03	12.6 ± 0.3	1.27 ± 0.21	0.33 ± 0.02	2.09 ± 0.09

### Statistical Analysis

Physicochemical parameters data were expressed as means of replicates based on a dry weight of compost materials. A pairwise correlation was conducted for each pair of variables including CH_4_ and N_2_O fluxes, bacterial gene abundance copies, and physicochemical parameters. Bacterial gene abundance copies and CH_4_ and N_2_O fluxes were log-transformed for normality and homoscedasticity as needed in statistical analyses. A linear stepwise regression model with the personality of Ordinary Least Squares (OLS) was used to fit CH_4_ and N_2_O fluxes by bacterial gene abundance copies and physicochemical parameters. In this method, regression variables are randomly selected based on prior probability, and the randomly selected variables are further screened by stepwise forward regression. Eventually, the forms of model are updated accordingly (**Table 3**). A *t*-test was used to examine the statistical significance of parameter estimates in the simulated OLS model.

**Table 3:** Modeling CH_4_ (Y_C_) and N_2_O (Y_N_) fluxes by coupling functional gene copy numbers with physicochemical parameters during windrow dairy manure composting and the model tested in rice paddies.

Biosystems	Model	k_1_		k_2_		k_3_		c		*R*^2^	RMSE	MEF
		Estimate	*P*	Estimate	*P*	Estimate	*P*	Estimate	*P*			
Manure composting	Y_C1_ = k_1_ ×*mcrA*/*pmoA* + c	3.05 ± 0.93	0.01					-1.39 ± 0.75	0.11	0.55	0.41	0.46
	Y_C2_ = k_1_× T + k_2_×*mcrA* + c	0.056 ± 0.006	<0.001	1.28 ± 0.13	<0.001			-9.06 ± 0.88	<0.001	0.94	0.14	0.94
	Y_C3_ = k_1_× T + k_2_×*mcrA* + k_3_× NH_4_^+^ + c	0.048 ± 0.04	<0.001	1.12 ± 0.08	<0.001	0.32 ± 0.08	0.009	-8.18 ± 0.51	<0.001	0.98	0.08	0.98
	Y_N1_ = k_1_× (*nirK*+*nirS*)/*nosZ* + c	1.40 ± 0.07	<0.001					0.06 ± 0.40	0.88	0.79	0.36	0.74
	Y_N2_ = k_1_×*nirK* + k_2_×*nosZ* + c	0.25 ± 0.04	<0.001	-0.87 ± 0.20	0.005			6.65 ± 1.40	0.003	0.92	0.23	0.90
	Y_N3_ = k_1_×*nirK* + k_2_×*nosZ* + k_3_×*pmoA* + c	0.20 ± 0.04	0.003	-0.77 ± 0.16	0.005	0.19 ± 0.08	0.07	4.93 ± 1.30	0.01	0.95	0.18	0.94
Rice paddy	Y_C3_ = k_1_× T + k_2_×*mcrA* + k_3_× NH_4_^+^ + c	0.02 ± 0.007	0.005	0.92 ± 0.14	<0.001	0.03 ± 0.01	0.005	-7.70 ± 1.12	<0.001	0.74	0.23	0.90
	Y_N3_ = k_1_×*nirK* + k_2_×*nosZ* + k_3_×*pmoA* + c	0.39 ± 0.08	<0.001	-0.35 ± 0.12	0.004	0.19 ± 0.07	0.02	-5.46 ± 0.99	<0.001	0.66	0.15	0.95

*The fluxes of CH_4_ and N_2_O and functional gene copy numbers were log-transformed for normality and homoscedasticity*.

### Model Test in Rice Paddies under T-FACE

To examine whether the simulated OLS models could also be applicable to other environmental systems, the models were tested against field measurements in paddy rice cropping systems under elevated atmospheric CO_2_ and rising temperature (a T-FACE platform). The field T-FACE platform was established in Kangbo village (31°300N, 120°330E), Guli Township, Changshu Municipality, Jiangsu, China, in 2010. The paddy field soil is a gleyic stagnic anthrosol formed on a clayey lacustrine deposit and cultivated under continuous rice–wheat rotation. The T-FACE platform had 12 octagonal plots, with the inner circle with an area of 25 m^2^ per plot. The experimental treatments included four experimental treatments with three replicates, namely, one with target atmospheric CO_2_ up to 500 ppmv (CO_2_), one with warming of canopy temperature by 1.5–2.0°C above ambient (T), and one with combined CO_2_ enrichment and warming (CO_2_+T), and taking an untreated plots with ambient condition as controls (Ambient). Seedlings of a local rice cultivar (Changyou 5) were transplanted into fields on June 20, 2014 and harvested on October 22, 2014. Spacing of hills was 15.3 × 25.4 cm (equivalent to 25.7 hills m^2^ and resulting in a plant density of 77.1 plants m^2^) for each experimental plots. All the field plots were under a typical water regime of flooding-midseason drainage-reflooding-moisture irrigation during the rice-growing season. The design of T-FACE platform and field experimental treatments and agricultural practice were detailed in Liu et al. (2014), Cai et al. (2015), and Chen et al. (2016).

In rice paddies, gas flux measurements and soil samples for physicochemical properties and microbial genes abundance analyses were simultaneously taken on July 6, July 21, August 12, August 30, September 10, September 24, and October 22, 2014. The CH_4_ and N_2_O fluxes were determined by the static chamber-GC method as shown in our previous studies (Zou et al., 2005; Liu et al., 2012), and the methods for gas flux measurements physicochemical properties and microbial genes abundance analyses were similar to those described in windrow composting experiment.

The three statistics *R*^2^ (coefficient of determination), RMSE (root mean square error), and MEF (modeling efficiency) were used for model evaluation (**Table 3**). All statistical analyses were performed using JMP software version 9.0.2 for Windows (SAS Inst., Cary, NC, USA, 2010).

## Results

**FIGURE 1:**
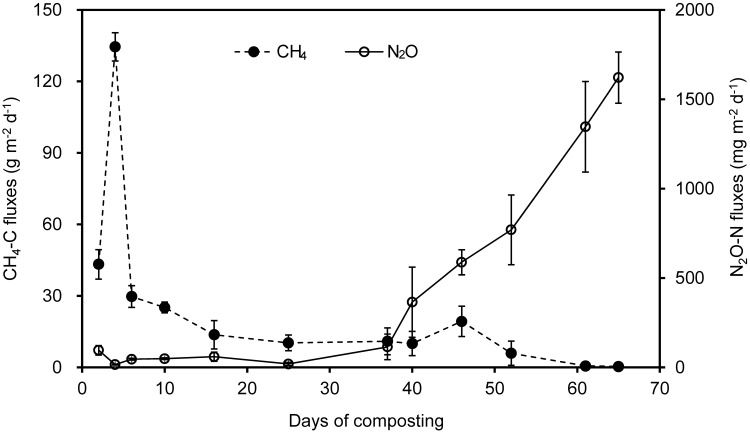
**Fluxes of CH_4_ and N_2_O during a 65-day period of windrow dairy manure composting**. Error bars show standard error of the mean of triplicate compost windrows.

There was a trade-off between CH_4_ and N_2_O fluxes during manure windrow composting (**Figure 1**). Substantial CH_4_ emissions occurred primarily during thermophilic Phase I. During Phase I, CH_4_ fluxes ascended rapidly until the peak fluxes were attained approximately 4 days after the onset of composting. Thereafter, CH_4_ emission was dramatically decreased by pile turning and then remained lower release rate, which was closely associated with decreases in pile temperature (**Table 2**). In contrast, N_2_O fluxes stayed relatively lower during Phase I, and they gradually increased during phase II. Eventually, N_2_O fluxes were highest by the end of manure compositing (**Figure 1**).

Over the 65-day period of manure composting, CH_4_-C fluxes varied from 0.4 to 134.5 g m^-2^ d^-1^, with an average flux of 17.1 g m^-2^ d^-1^ (**Figure 1**). Cumulative CH_4_ emissions in terms of initial windrow surface area were 1.1 kg m^-2^, being equivalent to 0.8% of total C in initial manure dry weight (MCF). The fluxes of N_2_O-N varied within the range of 15.0–95.0 mg m^-2^ d^-1^ during Phase I, and rapidly increased from 114.2 to 1621.1 mg m^-2^ d^-1^ during Phase II, dedicating to an average of 383.3 mg m^-2^ d^-1^ over the whole composting process. Cumulative N_2_O emissions in terms of initial windrow surface area were 25.1 g m^-2^, representing 0.18 kg per ton of MDW. The emission factor of N_2_O (EF, percentage of initial N in manure compost pile emitted as N_2_O-N) was estimated to be 1.2% for composting windrow.

**FIGURE 2:**
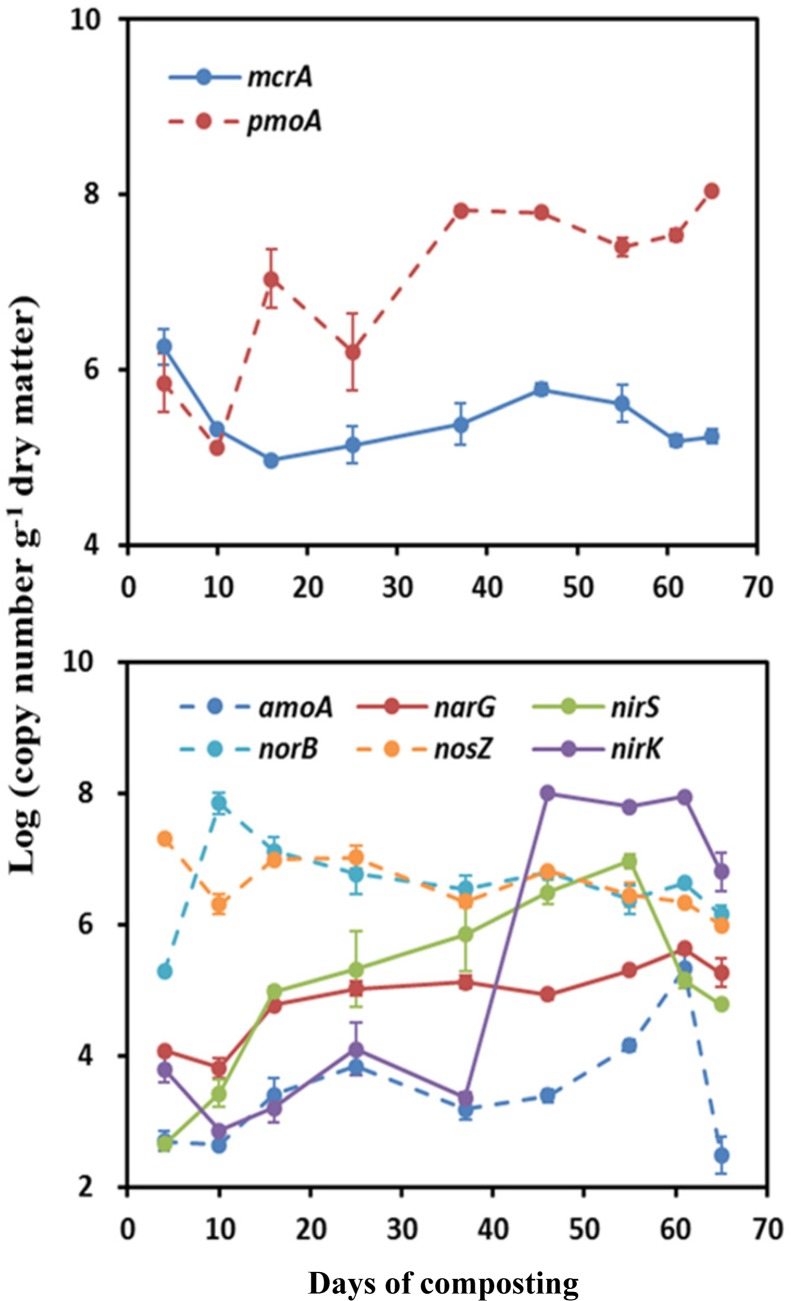
**Changes in functional gene copy numbers associated with CH_4_ and N_2_O emissions during windrow dairy manure composting**. Values indicate log-transformed gene copy numbers. Error bars show standard error of the mean of triplicate compost windrows.

A contrasting time course pattern of *mcrA* and *pmoA* genes abundance was detected during windrow composting. The measured abundances of *mcrA* were the highest at the onset of composting, being average 6.26 log copy numbers⋅g^-1^ (**Figure 2**). Thereafter, the *mcrA* gene abundance gradually decreased until it remained stable around ∼5.0 log copy numbers⋅g^-1^. Relative to a smaller variation of *mcrA* gene abundance, *pmoA* gene abundance showed larger variation (variation range: 5.11–8.05 log copy numbers⋅g^-1^) over the composting process. The measured abundance of *pmoA* decreased in the first week and then kept an ascending trend over the composting process, reaching the greatest abundance by the end of windrow composting (**Figure 2**).

For the functional genes involved in N_2_O emissions, the abundance of *amoA*, *narG*, *nirK*, and *nirS* genes shared a rising pattern over the composting process, in contrast to a declining trend for *norB* and *nosZ* genes abundance (**Figure 2**). Over the composting process, predominant genes in bacterial community shifted from *norB* and *nosZ* genes (6.76–6.91 log copy numbers⋅g^-1^) during Phase I to *nirK* gene (6.78 log copy numbers⋅g^-1^) during Phase II (**Figure 2**). The *amoA* gene abundance stayed the lowest (∼3.10 log copy numbers⋅g^-1^) over the whole windrow composting.

**FIGURE 3:**
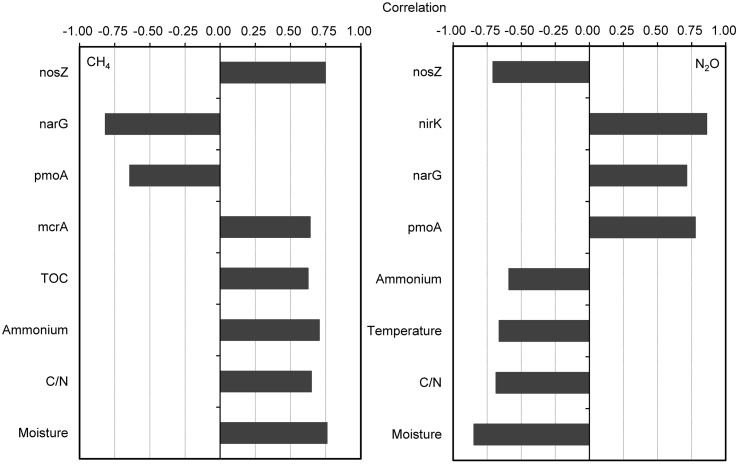
**Pairwise correlations among all tested variables showing significant probability (*p* < 0.05) for correlation of CH_4_ and N_2_O fluxes with physicochemical parameters and bacterial gene abundances**. The gene copy numbers and CH_4_ and N_2_O fluxes are log-transformed for normality and homoscedasticity.

The CH_4_ fluxes showed strong positive correlations with compost material parameters including moisture, C/N ratio, NH_4_^+^-N, and TOC during the composting process (**Figure 3**). The CH_4_ fluxes were also positively correlated with *mcrA* and *nosZ* gene numbers, but negatively correlated with *pmoA* and *narG* gene numbers. For the relative abundance of functional genes group, CH_4_ fluxes were positively correlated with *mcrA*/*pmoA* (*r* = 0.78, *p* = 0.01). Besides strong negative correlations between N_2_O fluxes and pile moisture, temperature and C/N ratio, N_2_O fluxes showed significant positive correlations with *pmoA*, *narG*, and *nirK* genes abundance, but negatively correlated with *nosZ* gene abundance (**Figure 3**). The N_2_O fluxes were positively correlated with relative abundances of functional gene group (*nirK*+*nirS*)/*nosZ* (*r* = 0.90, *p* = 0.001).

**FIGURE 4:**
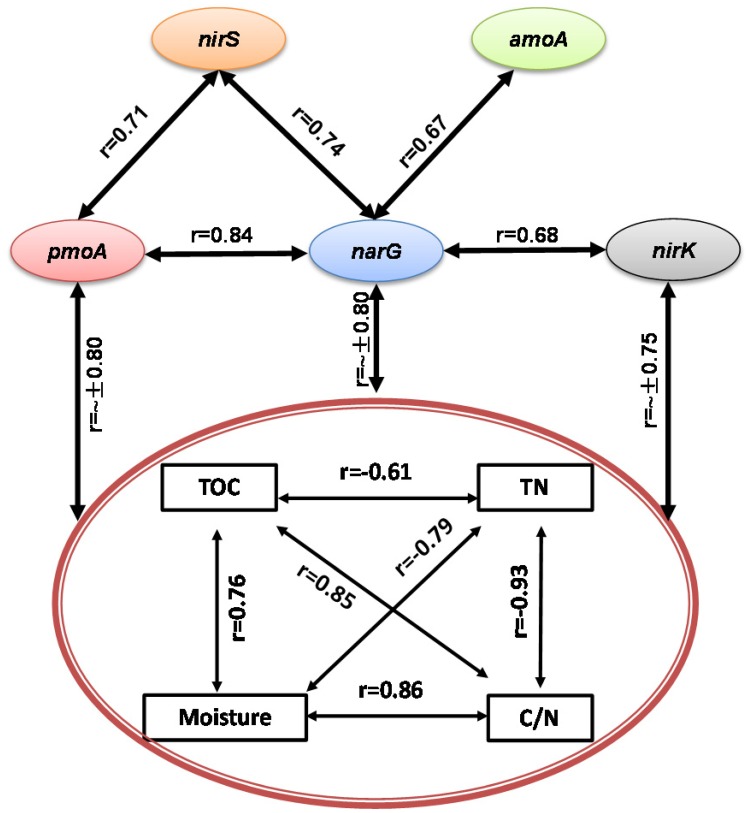
**Pairwise correlations among all tested variables showing some interacting physicochemical parameters significantly correlated with bacterial gene abundances (*p* < 0.05)**.

During the composting process, TOC, TN, moisture and C/N ratio were significantly correlated with each other, acting as a group to correlate with *pmoA*, *narG*, *nirK*, and *nirS* genes abundance and CH_4_ and N_2_O fluxes (**Figures 3**, **4**). Among functional genes, the abundance of *pmoA*, *narG*, and *nirS* genes were correlated with each other. Besides, the *narG* gene abundance was correlated with *amoA* and *nirK* genes, but *amoA* gene abundance was not significantly correlated with *nirK* gene (**Figure 4**).

**FIGURE 5:**
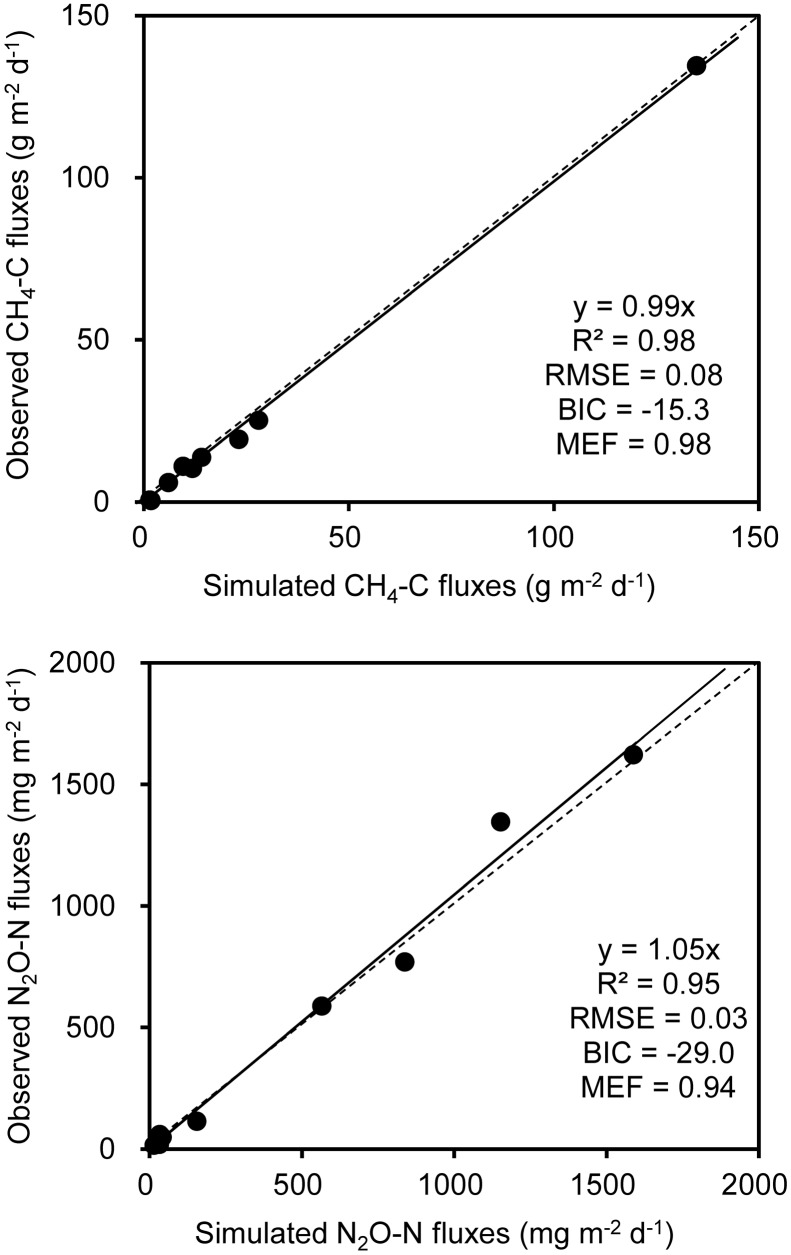
**Comparison of observed and modeled CH_4_ and N_2_O fluxes during dairy manure windrow composting**. Estimation of parameters in models for CH_4_ (Y_C3_) and N_2_O (Y_N3_) are shown in **Table 1**. The slope, *R*^2^, BIC, the root mean square error (RMSE), and model efficiency (MEF) are indicated at the bottom right of each panel.

A stepwise regression analysis was conducted for modeling CH_4_ and N_2_O fluxes with bacterial functional gene abundances and physicochemical parameters (**Table 3**). The model based on bacterial functional genes *mcrA/pmoA* alone can explain 55% of the variance in CH_4_ fluxes over the composting process (Y_C1_, **Table 3**). While integrating *mcrA* gene abundance together with pile temperature (T), however, the simulated regression model explained as high as 94% of the variance in CH_4_ fluxes (Y_C2_, **Table 3**). Furthermore, the regression model including *mcrA* gene, T and NH_4_^+^-N significantly lowered the model error and increased model efficiency, which can almost fully project the time course of CH_4_ fluxes (Y_C3_, **Table 3** and **Figure 5**). Among the regression models, the model based on pile temperature (T), *mcrA* and NH_4_^+^-N appeared to be the best fit for CH_4_ flux variance when the statistics *R*^2^, *P*, *RMSE*, and *MEF* were comprehensively evaluated (**Table 3**).

As shown in the stepwise regression model, a functional genes group, (*nirK*+*nirS*)/*nosZ* acted as a good proxy for predicting dynamics of N_2_O fluxes (Y_N1_, **Table 1**). While taking *nirK* minus *nosZ* genes into account but excluding *nirS* genes, the performance of simulated model was significantly improved, explaining as high as 92% of the variance in N_2_O fluxes during windrow composting (Y_N2_, **Table 1**). Besides *nirK* and *nosZ* genes, *pmoA* gene was also responsible for the variance in N_2_O fluxes as shown in pairwise correlation (**Figure 3**). Indeed, about 95% of the variance in N_2_O fluxes can be explained by the model based on linear regression of *nirK*, *nosZ*, and *pmoA* genes abundance (Y_N3_, **Table 1**). Compared to the Y_N1_ and Y_N2_ models, the Y_N3_ model including *pmoA* gene abundance as an additional predictor was able to minimize the uncertainty in N_2_O flux estimates (**Table 1** and **Figure 5**).

**FIGURE 6:**
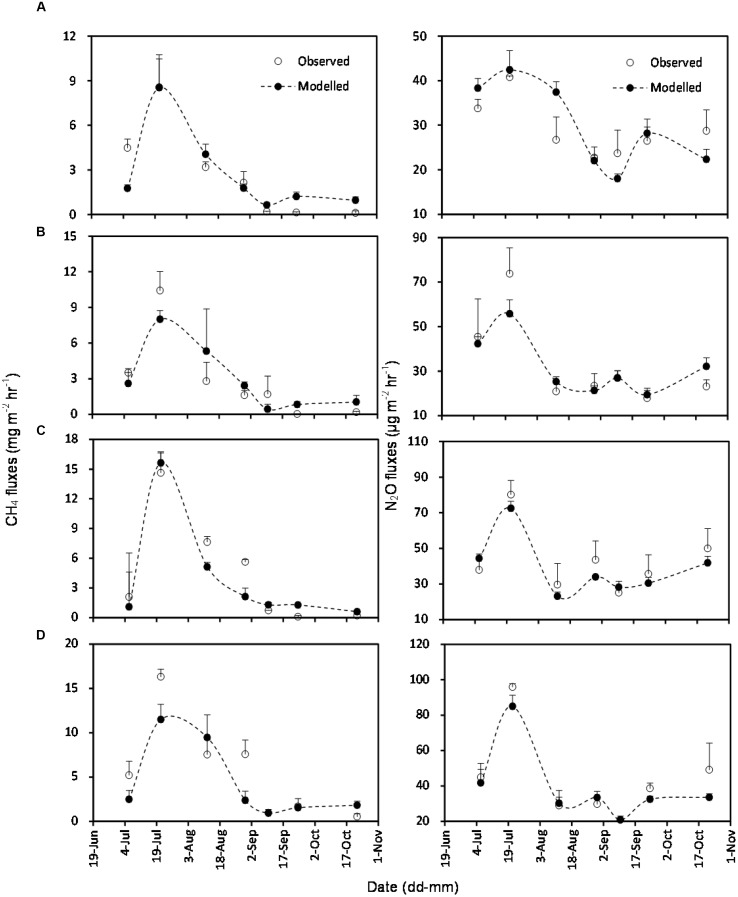
**Comparison of observed and modeled CH_4_ and N_2_O fluxes from paddy rice cropping systems under elevated atmospheric CO_2_ concentration and rising temperature (T)**. **(A)**, ambient; **(B)**, elevated CO_2_; **(C)**, rising temperature (T); **(D)**, elevated CO_2_ and rising temperature. Error bars show standard error of the mean of data. Overall statistics of model tests are shown in **Table 3**.

**FIGURE 7:**
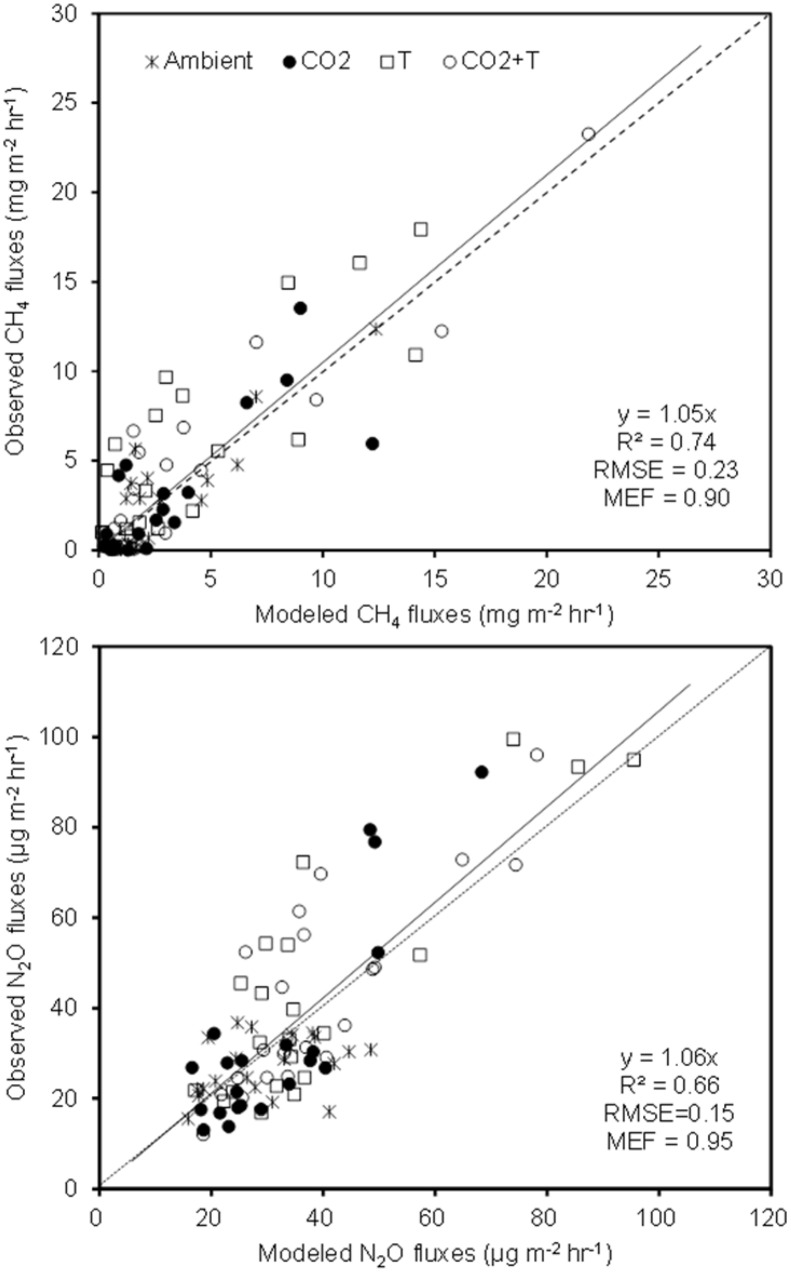
**The Y_C3_ and Y_N3_ models tested against field measurements in paddy rice cropping systems under elevated atmospheric CO_2_ and rising temperature (T)**.

The simulated OLS models were also applicable to paddy rice cropping systems (**Table 3** and **Figures 6**, **7**). In rice paddies, about 75% of the seasonal variance in CH_4_ fluxes as a response to elevated atmospheric CO_2_ concentration and rising temperature can be explained by re-parameterized Y_C3_ model based on linear combination of soil temperature, *mcrA* and NH_4_^+^-N (**Figure 7**). Similarly, the re-parameterized *nirK*, *nosZ*, and *pmoA* genes abundance in Y_N3_ model can largely reflect seasonal CH_4_ fluxes response to elevated atmospheric CO_2_ concentration and rising temperature in paddy rice cropping systems (**Figure 7**).

## Discussion

Manure composting has been recognized as an important source of CH_4_ and N_2_O to atmosphere [Intergovernmental Panel on Climate Change (IPCC), 2006; Chadwick et al., 2011; Hou et al., 2015; Owen and Silver, 2015; Pardo et al., 2015]. The IPCC guidelines introduce the terms of MCF (methane conversion factor) and EF (emission factor of N for N_2_O) for accounting CH_4_ and N_2_O emissions from manure composting, respectively [Intergovernmental Panel on Climate Change (IPCC), 2006]. In the present study, total CH_4_ and N_2_O emissions were estimated to be 1.1 kg m^-2^ and 25.1 g m^-2^, being equivalent to a MCF of 0.8% and an EF of 1.2% for composting windrow, respectively. The value of MCF in this study falls well within the IPCC default value range of 0.5–1.5% in composting windrows [Intergovernmental Panel on Climate Change (IPCC), 2006]. The estimated EF (1.2%) in this study is slightly greater than the IPCC default EF of 1.0% [Intergovernmental Panel on Climate Change (IPCC), 2006], but highly close to the recent estimates (mean of EF: 1.2%) based on a summary of available data on composting windrow by Pardo et al. (2015).

Measurements of GHG fluxes showed a trade-off between CH_4_ and N_2_O fluxes, as previously found in rice paddy soils and windrow compost (Cai et al., 1997; Hou et al., 2001; Zou et al., 2005; Ahn et al., 2011; Shen et al., 2011). Consistent with previous studies (Hou et al., 2001; Sánchez-Monedero et al., 2010; Sharma et al., 2011; Chen et al., 2014), substantial CH_4_ emissions occurred mainly in the early stage of manure composting process. In contrast, remarkable N_2_O emissions were triggered around the middle stage of the composting when pile temperature started to decline and oxygen availability was limited (Fukumoto et al., 2003; Xu et al., 2007; Ahn et al., 2011; Tsutsui et al., 2013; Wang et al., 2013). In addition, *norB* and *nosZ* genes were relatively predominant during the early stage of composting (**Figure 2**), suggesting that much N_2_O was further transformed into N_2_ as the final product in denitrification.

Consistent with the first hypothesis prediction, changes in physicochemical parameters shaped different time course patterns of CH_4_- and N_2_O-related functional genes (Gödde and Conrad, 1999; Holtan-hartwig et al., 2002; Kandeler et al., 2006; Zhou et al., 2011; Zhang et al., 2015). Correlation analyses showed that the abundance of *pmoA*, *narG*, *nirK*, and *nirS* genes shared negative correlations with pile temperature (*r* = ∼-0.70, *p* < 0.05), moisture, TOC, and C/N ratio, and positive correlations with TN (**Figure 4**), suggesting that changes in interacting physicochemical parameters in the pile shaped the dynamic pattern of the *pmoA*, *narG*, *nirK*, and *nirS* genes (**Figure 4**), in line with the results obtained by Hallin et al. (2009) showing that nitrate reducers and denitrifiers were closely related to soil TOC, TN, and C/N ratio in a 50-year-old fertilization experiment. Similar relationships between methanotrophs abundances and abiotic parameters were found in landfill cover soils (Kumaresan et al., 2009; Lin et al., 2009). Some studies also reported the relationships between denitrifiers and physicochemical parameters, such as correlations of *nirK* and *nirS* genes abundance with pile temperature (Zhang et al., 2015), and correlations of *narG*, *nirK* and *nosZ* genes abundance with soil TOC (Kandeler et al., 2006; Zhou et al., 2011).

Significant correlations of *nosZ* gene abundance with NH_4_^+^ and NO_2_^-^ suggested that *nosZ* gene abundance dynamics was mainly associated with substrate availabilities. The *mcrA* and *amoA* genes abundance did not show significant correlations with any of physicochemical parameters, and *amoA* gene abundance was, on average, much lower than nitrate reducers and denitrifiers (*narG*, *nirK nirS*, *norB*, and *nosZ*) abundance, suggesting that denitrification was much stronger than nitrification during windrow composting (Hao et al., 2001). Partially due to pH remaining stable around 8.0 during windrow composting, pile pH did not show significant correlations with any of bacterial functional genes in this study, consistent with Kandeler et al. (2006) but contrary to other previous studies (Deiglmayr et al., 2004; Bárta et al., 2010; Zhang et al., 2015). In addition, NH_4_^+^ was slightly correlated with *narG*, *nirK*, and *nirS* genes abundance (*p* = ∼0.07), while NO_3_^-^ did not show significant correlations with nitrate reducer and denitrifier abundances, which might suggest that nitrate in pile manure is not important for denitrifiers (Tiedje, 1988; Mergel et al., 2001; Avrahami et al., 2002; Liu et al., 2003; Kandeler et al., 2006; Zhang et al., 2015).

The NH_4_^+^ and *pmoA* gene were involved in CH_4_ and N_2_O emissions, respectively, which partially supported the second hypothesis that some specific physicochemical parameters and compositional bacterial enzymes encoded by relevant genes would be multifunctional as involved both in CH_4_ and N_2_O. The CH_4_ fluxes showed a positive correlation with NH_4_^+^, and NH_4_^+^ was selected as an indicator in the stepwise regression model (**Table 3** and **Figure 3**). A great many studies have revealed that NH_4_^+^ has an inhibitory effect on CH_4_ oxidization through either competition for methane monooxygenase or generation of toxic hydroxylamine and nitrite from ammonium oxidation (Steudler et al., 1989; Bosse et al., 1993; Dunfield and Knowles, 1995; Hanson and Hanson, 1996; Duan et al., 2013; Dam et al., 2014; Karbin et al., 2015), although stimulation effects or no effects of NH_4_^+^ on methanotrophs were reported in some other studies (Dunfield et al., 1995; Delgado and Mosier, 1996; Dan et al., 2001; Krüger and Frenzel, 2003; Shrestha et al., 2010; Hu and Lu, 2015). The CH_4_ fluxes were negatively related with *narG* but positively related with *nosZ* genes abundances, which might be due to the significant correlations of *narG* with *pmoA* genes (*r* = 0.84, *p* = 0.005) and of *nosZ* genes with NH_4_^+^ (*r* = 0.76, *p* = 0.02). In addition, N_2_O fluxes were correlated with *pmoA* gene abundances, and *pmoA* gene abundances were included in the regression model (**Table 3** and **Figure 3**), which might suggest denitrification with methane as external carbon source. Some studies reported that aerobic methane-oxidation coupled to denitrification is accomplished by aerobic methanotrophs oxidizing methane and releasing soluble organics that are used by coexisting denitrifiers as electron donors for denitrification (Modin et al., 2007). Indeed, the *pmoA*, *narG*, and *nirS* gene abundances were correlated in this study (**Figure 4**).

Both *mcrA* and *pmoA* genes abundances were correlated with CH_4_ fluxes, and the balance of *mcrA/pmoA* genes abundance was selected as a good indicator in the regression model (**Table 3** and **Figure 3**), indicating that both methanogens and methanotrophs played important roles in CH_4_ fluxes from composting windrow. The N_2_O fluxes were positively correlated with *narG* and *nirK* genes abundance, but negatively correlated with *nosZ* gene abundance. However, N_2_O fluxes were not correlated with *nirS* gene abundances in this study. The nitrite reducers with Cu-containing enzyme encoded by *nirK* gene are generally believed to be more important than those with cytochrome cd1 nitrite reductase encoded by *nirS* gene in the nitrite reduction step during manure composting (Yoshida et al., 2009; Bárta et al., 2010; Chen et al., 2010; Zhou et al., 2011; Zhang et al., 2015).

**FIGURE 8:**
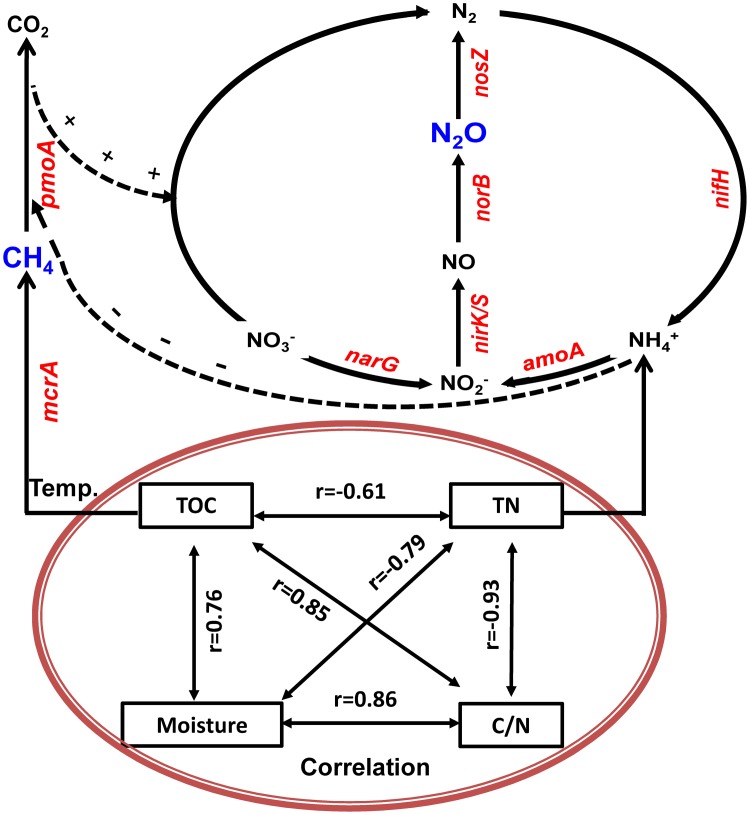
**Generalized schematic model showing predicted CH_4_ and N_2_O fluxes as a function of bacterial functional genes and physicochemical parameters during manure composting**. Physicochemical parameters of compost manure material including total organic carbon (TOC), total nitrogen (TN), moisture and carbon/nitrogen ratio (C/N) are correlated with each other, and interacting to shape the dynamics of bacterial functional gene abundance. Solid lines indicate direct connections. Dashed lines refer to indirect connections where ammonium has inhibitory effects on CH_4_ oxidization, while CH_4_ oxidization facilitates denitrification with methane as external carbon source.

Based on physicochemical and biological variables measurements during the composting and their correlation and regression analyses, we developed a schematic model that explains the dynamics of CH_4_ and N_2_O fluxes associated with bacterial functional genes and physicochemical parameters during manure composting (**Figure 8**). The schematic model shows how the prevalence of bacteria is involved in key steps in the process of CH_4_ and N_2_O emissions, and the CH_4_ and N_2_O fluxes during windrow composting are controlled by the interplay of enzyme encoding bacterial functional genes (**Figure 8**). In the schematic model, some physicochemical parameters are correlated with each other and interacting to shape the dynamics of bacterial functional gene abundance. Besides bacterial functional genes are directly involved in CH_4_ or N_2_O emissions, CH_4_ oxidization and denitrification processes interact together, where NH_4_^+^ has inhibitory effects on CH_4_ oxidization and *pmoA* gene abundance can facilitate denitrification with methane as external carbon source (**Figure 8**). Some studies stated that the aerobic methanotrophic bacteria are particularly useful for discovering and analyzing diverse mechanisms for nitrification and denitrification processes (Stein and Klotz, 2011; Zhou et al., 2014). By testing against samples in paddy rice cropping systems (**Table 3** and **Figures 6**, **7**), the simulated models can also be applicable to predicting seasonal dynamics of CH_4_ and N_2_O fluxes as responses to elevated atmospheric CO_2_ and rising temperature.

Bacterial genes key functional to CH_4_ and N_2_O fluxes identified in this study may be used as strategies for mitigating GHGs. For example, biochar application can significantly reduce both CH_4_ and N_2_O emissions by depressing *nirK* and *mcrA* while stimulating *nosZ* and *pmoA* genes abundances during manure composting (Sonoki et al., 2013; Wang et al., 2013). We noted that limitations on the use of qPCR for investigation of targeted genes also exist as results of PCR-bias, disturbance by DNA from dead cell, detecting only DNA copy numbers but not RNA transcriptional activity, lacking information regarding detailed community structures of specific microorganisms. The cDNA-based technologies and high throughout strategies, such as reverse transcription quantitative PCR, Illumina sequencing, and Gene Chip, will be very useful for a deeper understanding the characteristics of the functional genes and specific microbial groups, as well as their relationships with GHG emissions.

## Conclusion

We presented the quantitative study illustrating interactions between different bacterial activities and their role in controlling CH_4_ and N_2_O fluxes as a response to changes in physicochemical parameters during windrow composting. This study also presented the quantitative assessment of CH_4_ and N_2_O fluxes based on multiple microbial gene abundances at the functional levels in composting windrow. Additional studies in this area are highly needed to extend such capabilities and allow us to quantitatively address microbial contributions to GHG fluxes from soils and manure management systems. This is particularly important, as it is widely believed that microorganisms play important roles in global carbon and nitrogen biogeochemical cycles, yet they are generally not included in current biogeochemical models for carbon and nitrogen cycles.

## Author Contributions

SqL and JZ conceived this study. SqL has the main responsibility for microbial sampling and microbial analyses for this study. SwL and QS provided valuable input for the design and data analyses of this study. LS, XG, and YJ performed qPCR analyses and gas sampling. SwL, SqL, and JZ performed the statistical analyses and wrote the paper. All authors edited and approved the final manuscript.

## Conflict of Interest Statement

The authors declare that the research was conducted in the absence of any commercial or financial relationships that could be construed as a potential conflict of interest.
